# The role of social media for persons affected by infertility

**DOI:** 10.1186/s12905-020-00964-0

**Published:** 2020-05-24

**Authors:** Taina Sormunen, Klas Karlgren, Arthur Aanesen, Bjöörn Fossum, Margareta Westerbotn

**Affiliations:** 1 Department of Clinical Science and Education, Karolinska Institutet, Södersjukhuset, Stockholm, Sweden; 2 Department of Health Promoting Science, Sophiahemmet University, Stockholm, Sweden; 3 Department of Learning, Informatics, Management and Ethics, Karolinska Institutet, Stockholm, Sweden; 4 Department of health and functioning, Western Norway University of Applied Sciences, Bergen, Norway; 5 Sophiahemmet Hospital, Stockholm, Sweden; 6 Department of Nursing Science, Sophiahemmet University, Stockholm, Sweden

**Keywords:** Blogs, Experience, Infertility, Internet, Social media

## Abstract

**Background:**

Infertility remains a common universal disorder and a whole range of assisted reproductive technologies has been established. Society may fail to recognize the grief caused by infertility, which may lead to those struggling with it hiding their feelings. Previous research points out that infertile persons experience shortcomings in fertility care regarding continuity of care and social support. Social media may provide social and psychological support for infertile persons. Finding others who are going through similar experiences can help in the realization that the person is not alone and that her/his feelings are reasonable. The aim was to explore the roles of social media for persons affected by infertility.

**Methods:**

A cross-sectional, computer-assisted, self-administered online questionnaire, containing both open and closed questions, was used to collect data. The questionnaire was linked to the bulletin board of six closed infertility social forums. Both quantitative and qualitative analysis methods were used. A total of 132 participants completed the questionnaire containing questions about their use of social media dealing with infertility.

**Results:**

Most of the questionnaires were answered by females (97.7%) through Facebook (87%). Over 60% of the respondents had taken part in discussions about infertility in social media, between one and three years and 39% participated more than once a day. Half of the participants devoted one to three hours weekly to the forums and wrote 1–5 postings per week. The forums offered participants information, solidarity, and the opportunity to receive and give support. However, an adverse aspect that was described concerned advice that were not evidence-based. Infertility was experienced as being alienated from social life and being fragmented as a person.

**Conclusion:**

Participating in infertility forums offers persons information about fertility treatments and social support in the process of coping with infertility.

## Background

Parenthood is considered a social norm in society [1]. Therefore, infertility may call into question the most essential expectations persons have of themselves, their body, and their relationships. Discovery of impaired fertility may be followed by anger, guilt, depression and withdrawal [2] and infertility-related losses, such as loss of self-esteem, health, relationships and financial security [2].

Infertility remains a common global disorder and is estimated to affect between 8 and 12% of reproductive-aged couples [3]. In order to give couples affected by infertility the chance of having a child, a whole array of assisted reproductive technologies (ART), including in vitro fertilization (IVF), has been developed in the past 40 years [4]. Regardless of the cause of infertility in the couple, the female partner generally undergoes a multi-step fertility treatment [5]. Both involuntary childlessness and infertility treatment places psychological and emotional pressure on those involved [6].

Cousineau and Domar [7] highlight that persons faced with infertility experience a strong need of guidance and social support, a need that is not always met by existing sources of support. Previous research [8] points out that infertile persons experience shortcomings in fertility care, regarding continuity of care and social support and healthcare, may fail to recognize the grief caused by infertility. This may lead to increased feelings of shame and isolation and the persons may hide their feelings [9]. Finding others who are going through similar experiences can help the person to realize that she/he is not alone and that their feelings are reasonable. One way of doing this may be online social media, such as Facebook (FB) and blogs [10] which may offer persons with infertility a valuable source of support [11]. The aim of the study was to explore the roles of social media for persons affected by infertility.

## Methods

### Design

A cross-sectional study design was used. A self-administered online questionnaire consisting of both closed-ended and open-ended questions was utilized and directed at persons visiting online communities having a focus on infertility.

### Sample and setting

The first stage of the research process was to search for social media groups, with a focus on infertility issues, using search terms such as ‘infertility blog’, ‘Facebook’, ‘infertility’ and ‘involuntary childlessness’ in the Google search engine. The target population for this study was persons who identify themselves as infertile and who were members, readers or lurkers of online communities focusing on infertility.

Moderators of ten social media groups in *Vill ha barn* (in English, ‘I want a child’) and *Barnlängtan* (in English, ‘Longing for a child’) and eight closed Facebook groups focusing on infertility, were identified and contacted for permission to add a link to an online questionnaire from their bulletin boards. A cover letter containing information about the study background, aim, confidentiality, data collection and analysis method, was attached for full details of the study. Six out of ten moderators answered the request and agreed to add the link to their bulletin board. The recruitment letter and link to the web-survey were posted on the bulletin boards of the included forums. The questionnaire was based on a self-selected sample. For classification of the participants’ occupations, the Swedish standard of classification of occupations (SSYK) was used (Table 1).

**Table 1:** **Table 1** Presentation of the characteristics of the participants

Characteristics	Number of participants ***n*** = 132 (%)
***Gender***	
Female	129 (97.7%)
Male	3 (2.3%)
***Education***	
High school	53 (40.2%)
Higher education	77 (58.3%)
No answer	2 (1.5%)
***Profession***	
Managers, senior officials, legislators	7 (5.3%)
Professionals in various fields	21 (15.9%)
Associate professionals, technicians	16 (12.1%)
Clerks	18 (13.6%)
Service workers and shop sales workers	33 (25.0%)
Other professions	5 (3.8%)
Prefer not to answer	26 (19.7%)
No answer	6 (4.5%)
***Civil status***	
Heterosexual relation	121 (91.6%)
Single	5 (3.8%)
Same-sex relationship	5 (3.8%)
Prefer not to answer	1 (0.8%)
***Country of birth***	
Sweden	122 (92.4%)
Country outside Europe	6 (4.5%)
Other Nordic country	3 (2.3%)
Other European country	1 (0.8%)

### Online questionnaire

A 23-item online questionnaire was developed for this study using Google Forms, inspired by the study from Kaliarnta et al. [12]. The questions related to demographic characteristics, social media related behaviors and the functions of social media with a focus on infertility (Tables 1 and 2).

**Table 2:** **Table 2** Presentation of social media related behaviors of the participants (n=132)

Behaviour	Number of participants ***n*** = 132
***On which platform did you find this survey?***	
Facebook	*115 (87.1%)*
Barnlängtan (Longing for a child)	11 (8.3%)
Villhabarn.se (I want a child)	3 (2.3%)
Instagram	1 (0.8%)
No answer	2 (1.5%)
***How many years have you read and / or participated in blogs / Facebook groups / discussion forums about involuntary childlessness?***	
< 1 year	11 (8.3%)
1-3 years	80 (60.1%)
4-6 years	23 (17.4%)
7-9 years	15 (11.4%)
≥10 years	2 (1.5%)
No answer	1 (0.8%)
***How often do you take part in blogs / Facebook groups / discussion forums about involuntary childlessness?***	
More than once a day	52 (39.4%)
Once a day	28 (21.2%)
More than once a week	30 (22.7%)
Once a week	12 (9.1%)
Once a month	6 (4.5%)
Less than once a month	2 (1.5%)
No answer	2 (1.5%)
***How much time per week do you devote to blogs / Facebook groups / discussion forums about involuntary childlessness? (Estimate number of hours per week)***	
<1 hr	13 (9.8%)
1-3 hrs	66 (50.0%)
4-6 hrs	17 (12.9%)
7-10 hrs	22 (16.7%)
11-25 hrs	4 (3.0%)
>25 hrs	2 (1.5%)
No answer	8 (6.1%)
***On average, how many times a week do you write a posting on blogs / Facebook groups / discussion boards dealing with infertility?***	
No postings	53 (40.1%)
1 - 5 postings a week	66 (50.0%)
6 - 10 postings a week	7 (5.3%)
11 - 15 postings a week	3 (2.3%)
16 - 20 posting a week	1 (0.8%)
No answer	2 (1.5%)
***Do you use a pseudonym (fictitious name)?***	
Yes	17 (12.9%)
No	113 (85.6%)
No answer	2 (1.5%)

Matrix, multiple choice and open-ended essays or free-text questions were used and presented in the same order to all participants. Participants could move to the next question without providing an answer. A draft questionnaire was piloted as recommended by Polit and Beck [13]. By using snowballing, ten persons with a known history of infertility were recruited, answered, reviewed, and offered qualitative assessment allowing for refinements of the questions and they were not included in the study.

### Data collection

The data were collected during the fall of 2017. Persons, from the included social media groups, wishing to participate were directed to the online questionnaire where they were given additional information about the research project and their rights as participants, such as the possibility to stop participating in the study at any time. In total, 132 persons completed the online questionnaire.

### Analysis

Descriptive statistics were used for the analysis of the close-ended responses, utilizing Microsoft Excel Version 1908. Open-ended responses were analyzed qualitatively in an iterative process, using conventional inductive content analysis to develop sub-categories, categories and themes, inspired by Hsieh and Shannon [14]. The first author (TS) together with last author (MW) reviewed the text responses. The content was discussed and re-sorted until agreement was reached in the research team, as recommended by Sandelowski [15]. Quotations were selected to represent the main categories.

### Ethical considerations

The Swedish Research Council’s Guidelines for ethical assessment of medical research on humans have been followed during the entire research process [16]. Approval from the Ethical Review Board, Stockholm (*EPN Diar.nr.: 2015/2290–31/5*) was obtained. Answering the questionnaire was considered as giving informed consent to participation.

## Results

Participant characteristics are presented in Table 1. A total of 122 participants reported that their duration of infertility ranged between 1 to 25 years with a mean of 4.8 and a median of 4 years.

Results of the questions regarding social media behavior among participants are presented in Table 2.

Open-ended questions explored participants’ experiences regarding the role of social media, experiences from participating in social media and experiences of infertility. These descriptions were analyzed with qualitative content analysis and grouped into the main ideas.

Of the 132 participants, 125 answered the question: “What does participation in social media that focuses on infertility offer you?” Most of the responses were short, from one word to one sentence. A sentence could refer to several different roles. Using content analysis, four benefits were identified and quantified (Table 3). Solidarity, described as fellowship between the forum members, was highly valued. Furthermore, the participants described that those who have experienced infertility themselves, can best understand and explain different aspects of infertility.

**Table 3:** **Table 3** Benefits of social media

Benefits	N (%)
Solidarity	40 (32%)
Information	37 (30%)
To get and to give support	34 (27%)
Understanding infertility	14 (11%)
**Total**	N=125

A total of 104 participants answered the question about positive and negative experiences from participating in social media: 89 positive experiences and 15 negative experiences. When the participants were asked about what kind of experiences, they have from taking part in social media focusing on infertility, the answers seem to be a multitude of helpful aspects. The most prominent ones include feelings of fellowship with others in similar situations, solidarity and being a member of a tight community. Furthermore, women felt that they received invaluable information regarding fertility treatments, support to get through difficult times and hope for the future. Participants also defined negative experiences, such as becoming emotionally affected by negative treatment results or miscarriages of the forum members. Feelings of despair described by other forum members were experienced mentally straining by the participants. Jealousy for those who had successful treatments or gave birth to a child was also reported. Some women were disturbed by tips and advice that were not scientifically evidence based. Through social media the women could obtain understanding for their situation, which they sometimes lacked in the physical society.

In total, 118 participants answered the open-ended question regarding the impact of infertility. The free text responses provided information that was organized into four categories (Table 4). The main theme that emerged was *The experience of being fragmented and feelings of disconnectedness*.

**Table 4:** **Table 4** Presentation of subcategories and theme that emerged from the analysis

Subcategory	Category	Theme
Depression and anxiety Life’s sadness	Influence on the psychological health	The experience of being fragmented and feelings of disconnectedness
Life is on hold Loss of joy in life	Lacking meaning in life	
Female identity Partner relationship	Not complete as a woman	
Social isolation Envy of others	Alienation from social life	

### Influence on psychological health

The participants reported infertility as being anxiety-provoking. The stress caused, not only by infertility, but also from going through fertility treatments and by the prospect of treatments, induced *depression, and anxiety*. Experiencing infertility was the greatest grief they had in their lives and they felt desperate and had lost all hope. The participants described subjective symptoms of *depression* ranging from extended periods of crying to withdrawal from social life: “Infertility caused depression, anxiety, sick-listing, feelings of exclusion and it was a tough journey for our relationship.”

The participants described a wide range of distressing reactions as *life’s sadness* and distressing reactions, and one participant wrote: “Enormous grief over life not flowing as it should. Not knowing how the future is going to be, whether we are able to have children or not. As if we were left outside watching all our friends experience the happiness of being parents … when their baby is born, it is like a wall between our friends and us. As if we are standing outside a window and only allowed to look in through it, but not enter.”

### Lacking meaning in life

Several of the participants reported that infertility had caused a sense of meaninglessness and lack of goals in life. Some women stated that “their *life is on hold* and a longing for children occupies their whole life and it is eating me up from the inside … my life has come to a complete standstill.” The women also reported that they sensed a *loss of joy in life* and that they “were a shell of what they used to be” and felt different compared to other women.

### Not complete as a woman

The female participants reported that they viewed their body as “defective” and “unproductive”, which caused feelings of “*incompleteness as a woman*”. Additionally, some participants stated that infertility made them feel worthless and bitter: “Feelings shift between hope and despair, disappointment over the body that does not work as it should.” Another participant described her experiences like this: “You feel that you are medically handicapped. A large part of female identity vanishes”.

Some of the participants stated that infertility affected their sexual life negatively, as it was no longer experienced as romantic or pleasurable: “Sexual life becomes a routine thing … the *relationship* gets a blow when infertility makes you exhausted and sad.” For some women, support from the spouse was invaluable and together they could support each other: “The feeling that we can handle many setbacks and we are proud of what we have gone through..., to some extent exciting experiences of IVF.”

### Alienation from social life

The participants described the negative social consequences infertility had caused them. Many of them emphasized that they did *not like to participate in social activities* to avoid tough questions and expectations from others: “Infertility has for many years influenced my life in many ways, I experience that I have withdrawn and avoid friends and acquaintances who are pregnant or have children”. Some participants described *the envy* and the resentment they felt towards others who became pregnant or had a child: “Longing for a biological child, and the jealousy of seeing pregnant women and those with children.”

## Discussions

The aim of this study was to explore the roles of social media for persons affected by infertility and most of the participants were female, which is in accordance with a previous study [17]. Male participants are generally more difficult to recruit to research studies [18] about reproductivity [19]. The questionnaire was mostly answered through Facebook, which is understandable since it is the most popular social network in the world with more than two billion monthly active users [20]. In Sweden, 53% of the population uses Facebook daily [21]. To our knowledge the present study is one of few studies that have used closed social media with focus on infertility to collect data. Over half of the participants in our study had accessed social media with a focus on infertility, during one to three years, and four out of ten partook more than once a day. Half of the participants devoted one to three hours weekly to the forums and wrote between one to five postings per week. Our finding are in line with the results from the study of Kahlor et al. [11] that the perceived benefits of the forums for the participants were experiencing solidarity, exchanging information and the possibility to receive and give support. In our study, infertility was described by the participants as being fragmented as a person and being alienated from social life. In other words, social media may be used to process the emotional side of infertility, as stated by Malik and Coulson [22], rather than finding information about factual medical care. Barker’s findings show that online social media groups can provide information that cannot be found anywhere else [23], because other forum members are in similar situations and have been through the experience of the disease. Health professionals tell patients only what they know as a professional or what they feel the patient needs to know [24].

On Facebook, persons can be identified by applications, protocols, and tools, such as Internet protocol (IP) addresses and cookies. This information can be used to find out more about, and identify, people [25]. Infertility is considered a sensitive issue by many, and therefore some potential participants chose not to participate in this study, due to fear of being identified and to protect their identity. Mierlo [26] explains that lurking is a common behavior found in numerous content providing sites, and in this study nearly half of the participants identified themselves as lurkers. Hannon et al. [27] explain that lurkers are silent/invisible participants, who contribute very little or no content and choose to participate in the forum quietly. Malik and Coulson [22] report that the main reasons for both lurkers and posters to visit an online social media group were to get information and to find persons in similar situations, which is in line with our results. Uden-Kraan et al. [28] point out that both posters and lurkers derive similar benefits from participating in health-related online social media.

Patel et al. [29] propose that online forums are likely to improve chronic disease care by providing emotional and social support. Van Empel et al. [8] state that infertile persons experience shortcomings in fertility care regarding social support. In the present study, the participants described that one of the most important roles of infertility forums was to enable them to receive and to give support. Our previous study has shown that some women are not able to discuss infertility-related subjects with their spouse [17] and therefore turning to infertility forums may be a way to process emotions and relations. A multitude of helpful aspects were mentioned regarding participation, such as being a member of a tight community and feelings of belonging and being surrounded by persons with similar experiences.

Results from the present study disclosed that one of the disadvantages of participating in the infertility forums was becoming emotionally affected by other members’ negative experiences. Further, some participants described that some information given in the forums was inaccurate. Forum members can have limited knowledge of the medical history of the other members and therefore the advice and information given may not be adequate in every case [30].

Finally, the present study shows that persons affected by infertility experience feelings of fragmentation and alienation, and social media offers them opportunities to process their infertile situation, to receive information and support, which has been shown also in previous studies [10, 11]. A U.S. study investigating online infertility forums showed that infertility was experienced as a stigma but also that many forum posts indicated that infertile women attempted to reverse stigma power by stigmatizing their fertile friends by referring to them as overly fertile [31]. This study similarly showed how women experienced negative social consequences of their infertility but while they described strong envy towards fertile friends the responses in this study did not show attempts at stigmatizing them.

Four out of ten forums did not respond to our request and one reason might be to protect the identity of forum members. It is difficult to determine a response rate for the study because we do not know how many forum members saw the information about the online questionnaire and decided not to participate. Nonresponse bias is a threatening factor for the validity of the study results [32]. Massey and Tourangeau advocate the view that surveys are generally facing declining response rates in many societies [33] and also in Sweden [34].

Furthermore, most participants were women; the results are therefore not generalizable to men who may have other experiences and needs for support [35]. The findings are based on the participants’ views at one point in time and the study was conducted in a Swedish context and may therefore not be generalizable to other cultures. Most of the participants (58%) had a higher education. Hoybye et al. [36] state that participants in online social media forums tend to have a high level of education.

In online surveys there is a risk of multiple responses from the same participant. In the study, the option to block multiple responses from a single IP address was not used. However, the IP addresses of all participants were reviewed, showing that the survey was not completed from the same IP address on multiple occasions.

It is important to realize that 40% of the respondents in this study were lurkers, who can be difficult to reach. Zillman et al. [37] point out that respondents with high topic interest are more likely to participate in surveys. Between-group comparisons were not made, because most of the answers were sent via Facebook and the other groups were small, making comparisons difficult.

## Conclusion

Infertility was described as feeling disconnected from society. Participation in online media, in this study, is mostly experienced as positive. There are numerous roles of online media for infertile persons and they have various reasons for using these forums. The key benefits were solidarity, to receive and to give support and to understand the infertile situation. One of the adverse aspects was becoming emotionally affected by the experiences of other forum members.

## Data Availability

Due to confidentiality and risk for identification of participants the data is not shared. De-identified data can be made available upon reasonable request.

## Abbreviations

AA: Arthur Aanesen
ART: Assisted reproductive technologies
BF: Bjöörn Fossum
EPN: The Ethical Review Board
FB: Facebook
IVF: In vitro fertilization
KK: Klas Karlgren
MW: Margareta Westerbotn
SSYK: The Swedish standard of classification of occupations
TS: Taina Sormunen
U.S.: The United States
